# Effects of the inclusion of a mixed *Psychrotrophic* bacteria strain for sewage treatment in constructed wetland in winter seasons

**DOI:** 10.1098/rsos.172360

**Published:** 2018-04-25

**Authors:** Meizhen Tang, Zhengtao Li, Yuewei Yang, Junfeng Chen, Jie Jiang

**Affiliations:** College of Life Science, Qufu Normal University, Shandong, Qufu 273165, People's Republic of China

**Keywords:** mixed *Psychrotrophic* bacteria strain, constructed wetlands, wastewater treatment, kinetics mode

## Abstract

Constructed wetlands (CWs) have been used globally in wastewater treatment for years. CWs represent an efficient ecological system which is both energy-saving and low in investment for construction and operational cost. In addition, CWs also have the advantage of being easy to operate and maintain. However, the operation of CWs at northern latitudes (both mid and high) is sometimes quite demanding, due to the inhibitory effect of low temperatures that often occur in winter. To evaluate the wastewater treatment performance of a culture of mixed *Psychrotrophic* bacteria strains in an integrated vertical-flow CW, the removal rates of ammonia nitrogen (NH_3_–N), chemical oxygen demand (COD), nitrite nitrogen $\left({\text{NO}}_{2}^{-}-\text{N}\right)$, nitrate nitrogen $\left({\text{NO}}_{3}^{-}-\text{N}\right)$ and total phosphorus (TP) were quantified at different bacterial dosages to determine the best bacterial dosage and establish kinetic degradation models of the mixed strains. The bacterial culture was made up of *Psychrobacter* TM-1, *Sphingobacterium* TM-2 and *Pseudomonas* TM-3, mixed together at a volume/volume ratio of 1 : 1 : 1 (at bacterial suspension concentrations of 4.4 × 10^9^ ml^−1^). Results showed that the organic pollutants (nitrogen and phosphorus) in the sewage could be efficiently removed by the culture of mixed *Psychrotrophic* bacteria. The optimal dosage of this mixed bacteria strain was 2.5%, and the treatment efficiency of COD, NH_3_–N, ${\text{NO}}_{2}^{-}-\text{N}$, ${\text{NO}}_{3}^{-}-\text{N}$, total nitrogen and TP were stable at 91.8%, 91.1%, 88.0%, 93.8%, 94.8% and 95.2%, respectively, which were 1.5, 2.0, 2.1, 1.5, 2.2 and 1.3 times those of the control group. In addition, a pseudo-first-order degradation model was a good fit for the degradation pattern observed for each of these pollutants.

## 1. Introduction

A constructed wetland (CW) is an artificial wetland built to imitate a natural wetland ecosystem. Because of the low initial investment in construction, low operational costs, limited required maintenance, high efficiency and good compatibility of stable treatment effect and ecological landscape, CWs are widely used in processing agricultural sewage, acid mine drainage, industrial wastewater, aquaculture wastewater, landfill leachate and city highway runoff and drainage [1–8]. In recently published studies, it was found that the contaminant removal rate by CWs could reach 85% to 95% for biochemical oxygen demand (BOD), 80% for chemical oxygen demand (COD) and 92.41% for nitrogen compounds [9]. The main mechanisms of pollutant removal in constructed wetlands involve several biological processes (e.g. microbial metabolic activity and plant uptake) and physico-chemical processes (e.g. sedimentation, adsorption and precipitation at water-sediment, root-sediment and plant–water interfaces) [10]. Microbial degradation plays a dominant role in the removal of soluble/colloidal biodegradable organics in wastewater [11,12]. However, the sewage treatment efficiency of the constructed wetlands in northern China is rather low in winter. This is because microbial activity is reduced as the water temperature decreases, and (due to winter plant death) the biological activity of CWs might decrease under winter conditions. Thereby the pollutant removal efficiency and, more importantly, the efficacy of this contaminant removal technique might be affected by seasonal changes.

There is some global research on low-temperature biological strengthening techniques that can be applied to artificial wetland systems. Pei *et al*. [13] studied the impact of denitrification efficiency by *Bacillus subtilis* FY99-01 in wetland systems, and found that the microorganisms could effectively increase the removal of nitrates from the wetlands. Shao *et al*. studied the denitrification effects of *Paenibacillus* sp. XP1 dosing on cattails and reed surface flow in a CW system when temperature was between 15 and 21°C and found that *Paenibacillus* sp. XP1 introduction could effectively shorten the treatment time required for wastewater decontamination. Furthermore, after treatment with these bacterial cultures the concentration of ammonia (NH_3_–N) in effluent met the national level B standard in the CW, and compared with the reed CW, was more efficient at denitrification [14]. These results confirmed the feasibility of artificial wetland wastewater treatments at low temperatures [15]. The sewage treatment efficiency of *Pseudomonas flava* WD-3 in an integrated vertical-flow constructed wetland (IVCW) during winter with different dosages, *P. flava* WD-3 has a good processability for the wastewater treatment in the IVCW system in winter. Besides, the simplified Monod model simulated and evaluated the pollutant removal efficiency of this bacterial strain with respect to its dosages to improve water quality, and even accurately predicted the pollutant removal efficiency [16].

There are relatively few recent studies that have focused on the use of biological strengthening technology to increase the efficiency of the wastewater treatment in CWs. Owing to the effects of the natural environment, the applications of low-temperature biological reinforcement technology in CW systems still needs further research.

In this study the cold-resistant mixed bacteria cultures of *Psychrobacter* TM-1, *Sphingobacterium* TM-2 and *Pseudomonas* TM-3 (mixed *Psychrotrophic* bacteria strain), isolated from the Nansihu Lake wetlands, were used in the IVCW system. The removal rates of chemical oxygen demand (COD), ammonia nitrogen (NH_3_–N), nitrate nitrogen $\left({\text{NO}}_{3}^{-}-\text{N}\right)$, nitrite nitrogen $\left({\text{NO}}_{2}^{-}-\text{N}\right)$, total nitrogen (TN) and total phosphorus (TP) were measured to test the biotreatment performance of this mixed *Psychrotrophic* bacteria strain. The main objective of this paper was to develop a simplified first-order kinetics model that can be used with CW data to (1) predict COD, NH_3_–N, ${\text{NO}}_{3}^{-}-\text{N}$, ${\text{NO}}_{2}^{-}-\text{N}$, TN and TP retention in the wetland system, (2) evaluate the effects of different dosages and (3) provide a modelling tool to simulate and evaluate the pollutant removal efficiency of this mixed *Psychrotrophic* bacteria strain. The results of this study provide a theoretical foundation and scientific support for the use of constructed wetlands in wastewater treatment during the winter months, and may have great significance in solving the increasingly severe water pollution problem in China.

## 2. Material and methods

### 2.1. Isolation, screening and identification of the mixed *Psychrotrophic* bacteria strain

#### 2.1.1. Enrichment culture, separation, screening and purification of the *Psychrotrophic* bacteria

Sediment samples, collected from Nansihu Lake in Shandong province, were incubated at a temperature of 2°C for 7 days in order to domesticate the microbes. Five grams of sediment was placed in each conical flask and shaken in an orbital shaker incubator (150 r.p.m.) at a temperature of (6 ± 1)°C. To allow the microbes to proliferate rapidly, we placed a few vitreous balls and 95 ml of sterile culture medium in each flask.

One millilitre of the nutrient solution was added to each test tube with 9 ml of sterilized water (1 : 10 dilution of the culture medium). In this way, dilution to 10^−2^, 10^−3^, 10^−4^, 10^−5^ and 10^−6^ times less than the original concentration of the culture medium could be easily obtained. Subsequently, 1 ml of the solution was taken from each flask and diluted 10^−4^, 10^−5^, 10^−6^ times less than the original concentration. These solutions were then inoculated in three kinds of isolation media (with three parallels for each kind of medium), using agar plates. After that, the plates were placed into the incubator at a temperature of 6(±1)°C and growth was recorded constantly. The nine best-growing strains were selected and repeatedly purified to acquire a single colony. Then the colonies were inoculated in a slant culture medium and reserved in the refrigerator at 4°C.

#### 2.1.2. The screening of the mixed *Psychrotrophic* bacteria

After the enrichment cultivation, the nine single colonies were inoculated in the simulated wastewater while at a pH of 7.0. Inoculation quantity was controlled to within 10%. The solutions were statically cultured following 6 h of aeration, and the concentrations of COD, TP and NH_3_–N were measured every 12 h. According to the removal efficiency of the pollutants, the most efficient strains (1, 2, 3, 4 and 5) were selected.

These five bacterial strains, which had high-efficient degrading capability, were inoculated in lysogeny broth (LB) culture medium at different concentrations. The growth status of the mixed flora was recorded regularly. Meanwhile, the mixed flora was inoculated in the simulated wastewater at a pH of 7.0. The inoculation quantity was controlled within 10%. The solutions were then statically cultured after 6 h of aeration at 6(±1)°C, and the concentrations of COD, TP and NH_3_–N were measured every 12 h. According to the removal efficiency of the pollutants, the combination of the 1, 4 and 5 strains had the most notable effect on sewage disposal efficiency.

#### 2.1.3. Identification of the mixed *Psychrotrophic* bacteria species

To identify the species of bacteria in the mixed *Psychrotrophic* bacteria, the 1, 4 and 5 strains were dyed, placed under the microscope and the morphological characteristics of these bacteria were recorded in table 1. A preliminary identification was carried out using physiological and biochemical characteristics of the bacterial strains.

**Table 1.** Morphologic, physiological and biochemical characteristics of the bacterial strains 1, 4 and 5.

identification item	Strain 1	Strain 4	Strain 5
colony shape	translucent, round	opaque, round	translucent, irregular
colony colour	milky	milky	yellow
colony state	moist, neat edge	wet, irregular edges	moist, neat edge
microbial category	bacterial	bacterial	bacterial
Gram staining	+	−	+
gelatin hydrolysis	−	+	+
oxidase (V-P) assay	−	−	+
membrane experiments	+	−	+
oxidation and fermentation of glucose	+	−	+
oxidation and fermentation of sucrose	−	−	−
oxidation and fermentation of lactose	+	−	+
methyl red experiment	+	−	+
citric acid experiment	+	+	−
H_2_S production experiment	−	−	+

Using a scanning electron microscope the 1, 4 and 5 strains were purified and inoculated on an LB solid culture medium. After incubation at 30°C for 24 h, the 1, 4 and 5 strains were selected, isolated and then rinsed several times with sterilized water. Firstly, the thalluses were fixed with 3% glutaraldehyde, then rinsed, and fixed with osmic acid. Finally, the bacteria were dehydrated with ethanol.

16S rDNA sequence analysis: the total DNA of strains 1, 4 and 5 was extracted. Using the following primers: 8f (5′AGAGTTTGATCCTGGCTCAG 3′) 20 bp and 1492r (5′ GGTTACCTTGTTACGACTT 3′), PCR was performed. Following cloning and purification, the PCR product was identified by Shanghai Biological Engineering Co, Ltd [17–19].

### 2.2. Integrated vertical-flow constructed wetland systems and operation

**Figure 1.**
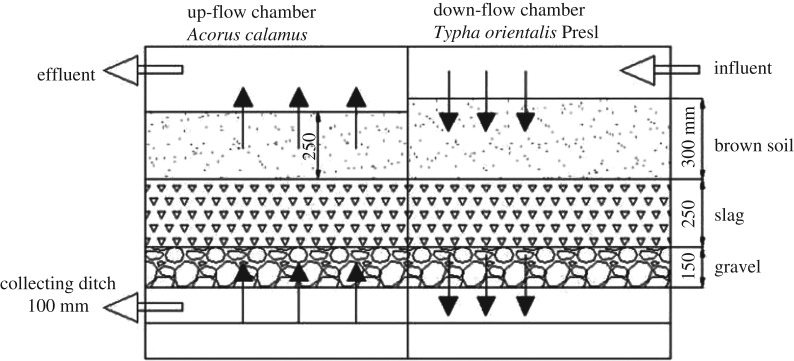
Schematic diagram of the IVCW system.

Six small-scale plots were constructed in March 2012. Each plot (2 m^2^) was equally divided into two chambers: a down-flow chamber and an up-flow chamber, indicating the direction of the passing water. A collecting ditch, 100 mm deep, was installed at the bottom of the chambers. Each chamber comprised three different particle-size-distribution layers: 150 mm depth of gravel (40–50 mm diameter) on the bottom, 250 mm slag (5–10 mm diameter) in the middle and 300 mm (in the down-flow chamber) or 250 mm (in the up-flow chamber) brown soil (0–4 mm diameter) on the top. All the water pipes were made of stainless steel (75 mm diameter). For an even distribution of water, the influent pipes had holes (5 mm diameter) on the underside and were spread on the surface of the down-flow chamber. At the bottoms of the two chambers, the collecting pipes transported water coming from the down-flow chamber to the up-flow chamber. The effluent pipes were situated on the surface of the up-flow chamber (figure 1). *Typha orientalis* Presl and *Acorus calamus* were planted in the down-flow and up-flow chambers. The dosing load of the CW was 2–20 cm d^−1^ and the organic load was 15–20 kg ha^−1^ h^−1^. When the system worked, the sewage was first dosed evenly on the surface of the down-flow chamber, then the sewage flowed downward vertically, finally reaching the up-flow chamber through a connecting pipe. After reaching the up-flow chamber, it flowed upward vertically, and finally was discharged from the effluent pipes. In this experiment, the CW was designed to be a discontinuous-flow system and was operated in a non-saturated state. The detention time in the system was set to be 5 days.

The influent water was taken from the sewage treatment plant in Qufu. Parameters are listed in table 2.

**Table 2.** Parameters of inflow of the pH and temperature. DO, dissolved oxygen; COD, chemical oxygen demand; TSS, total suspended solids; TP, total phosphorus; NH_3_–N, ammonium nitrogen; TN, total nitrogen.

index	pH	temperature (°C)	DO (mg l^−1^)	COD (mg l^−1^)	TSS (mg l^−1^)	TP (mg l^−1^)	NH_3_–N (mg l^−1^)	${\text{NO}}_{3}^{-}-\text{N}$ (mg l^−1^)	${\text{NO}}_{2}^{-}-\text{N}$ (mg l^−1^)	TN (mg l^−1^)
influent	7.2 ∼ 7.8	6 ∼ 10	3.4 ∼ 3.8	480 ∼ 500	2.1 ∼ 2.5	12.7 ∼ 13.8	43.3 ∼ 47.4	30.1 ∼ 37.1	2.0 ∼ 2.5	82.5 ∼ 84.5
effluent	7.0 ∼ 7.4	7 ∼ 10	0.2 ∼ 0.4	—	—	—	—	—	—	—

Preparation of bacterial suspension: the 1, 4, and 5 strains were incubated in the LB liquid culture medium until their absorbance reached 1.2. The solution was then centrifuged, then the supernatant was discarded and the bacteria thallus was diluted with a saline solution. This process was repeated three times so that the nutrient of the medium could be fully removed. In the end, the absorbance of the solution was adjusted to 1.2 with the saline water. These bacterial solutions were then mixed together at a volume ratio of 1 : 1 : 1.

In early December 2014 (water temperature between 4 and 10°C), suspensions of mixed *Psychrotrophic* bacteria (4.4 × 10^9^ ml^−1^) were injected into the IVCW system at a 0.5–5% volume/volume ratio to the sewage. We also included a control treatment that was not incubated with any *Psychrotrophic* bacteria. The concentrations of different parameters in effluent, including COD, NH_3_–N, ${\text{NO}}_{3}^{-}-\text{N}$, ${\text{NO}}_{2}^{-}-\text{N}$, TN and TP, were monitored constantly for three months.

All the parameters were analysed according to standard methods. Statistical analysis was performed using Origin8.6 and SPSS19 software.

## 3. Results and analysis

### 3.1. Identification of mixed *Psychrotrophic* bacteria

**Figure 2.**
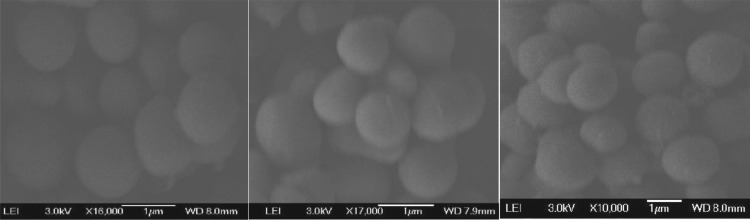
SEM image of strains 1, 4 and 5.

Identification results of morphologic, physiological and biochemical characteristics of the bacterial strains at 6(±1)°C can be found in figure 2.

**Figure 3.**
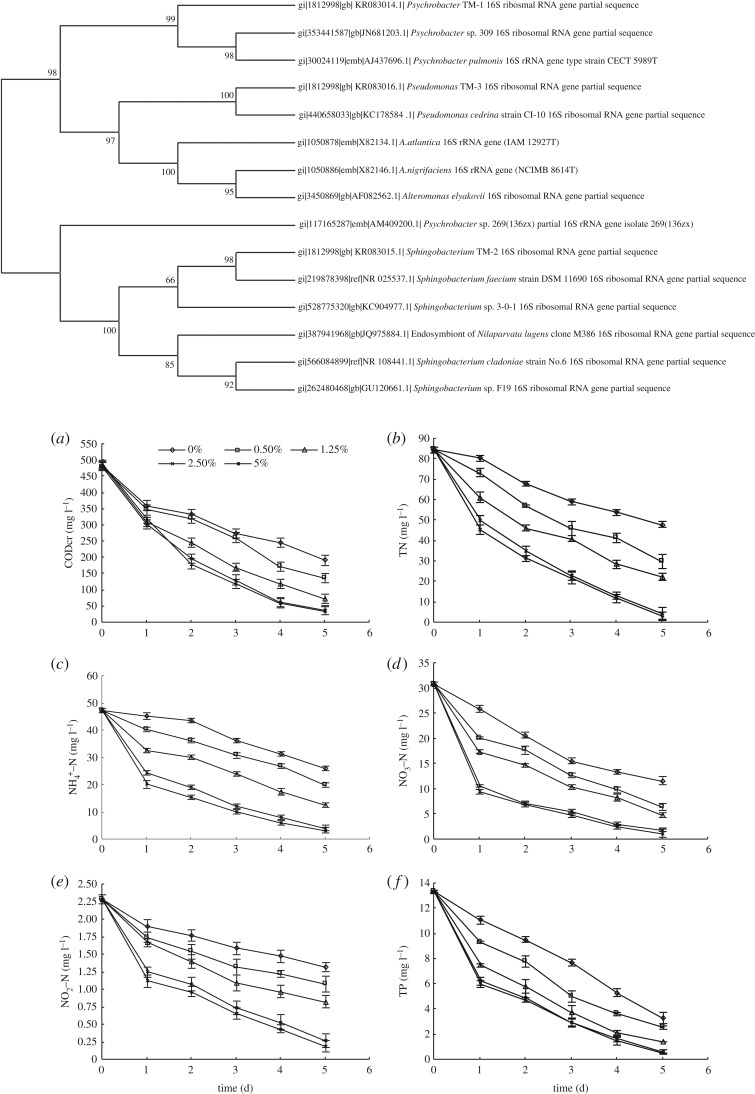
Phylogenetic tree of the bacterial strains 1,4 and 5. The concentration of COD, TN, NH_3_–N, ${\text{NO}}_{3}^{-}-\text{N}$, ${\text{NO}}_{2}^{-}-\text{N}$ and TP changes with time in the CW (as figure *a*, *b*, *c*, *d*, *e* and *f*).

Under cold-temperature conditions (6(±1)°C), Strain 1 was composed of a Gram-positive translucent circular ivory colony, with a moist surface, and regular edge. Strain 4 was a Gram-negative opaque round milky colony with a moist surface, and irregular edge. Strain 5 was a Gram-positive translucent yellow colony with a moist surface and irregular edges. Results from SEM (scanning electron microscopy) are shown in figure 2. Strains 1, 4 and 5 were are all spherical with no flagella. According to the Shanghai Biological Engineering Co., Ltd, the specific 16S rDNA sequence of Strain 1 was 1498 bp (GenBank acceptance number KR083014), the sequence of Strain 4 was 1489 bp (GenBank acceptance number KR083015), and the sequence of Strain 5 is 1489 bp (GenBank acceptance number KR083016). A BLAST search was performed on the three strains, and the phylogenetic tree was analysed and constructed by using Clustal W and PHYLIP software. The phylogenetic tree is shown in figure 3.

According to the physiological and biochemical characteristics and the 16S rDNA of strains, strains 1, 4 and 5 could be preliminarily identified as cold *Bacillus*, sphingolipids of *Bacillus* and *Pseudomonas*. Strain 1 was named *Psychrobacter* TM-1, and strains 4 and 5 were named *Sphingobacterium* TM-2 and *Pseudomonas* TM-3, respectively.

### 3.2. Effect of simulated wastewater treatment

The degradation efficiencies of COD, NH_3_–N and TP from the simulated sewage at 6(±1)°C by these mixed *Psychrotrophic* bacteria cultures were 86.83%, 65.95% and 56.08%, respectively. Average removal efficiencies were 1.38, 1.17 and 1.11 times higher than the single strains of *P. flava* WD-3 (GenBank acceptance number JX114950) [20]. In addition, the removal performance was found to be very stable.

### 3.3. The effect of sewage treatment on an artificial wetland

**Figure 4.**
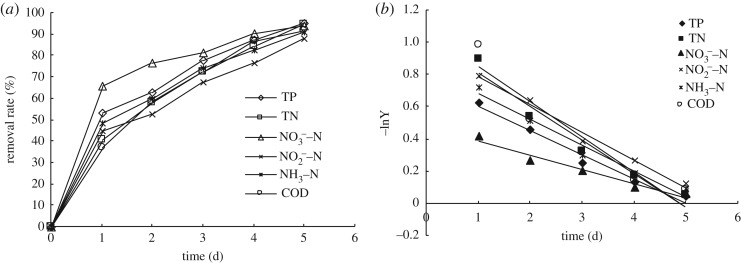
The removal rates of COD, TN, NH_3_–N, ${\text{NO}}_{3}^{-}-\text{N}$, ${\text{NO}}_{2}^{-}-\text{N}$ and TP changes with time when dosage is 2.5%.

When the hydraulic retention time (HRT) was 5 days and the temperature of water was 6–10°C, the water treatment efficiency in the CW with different inoculation amounts of low-temperature mixed flora was as shown in figures 3 and 4.

As shown in figure 3, between 0.5% and 5.0% bacteria the purgative efficiency of sewage in wetland system increased as the dosage of the mixed *Psychrotrophic* bacteria was increased. Indeed, when the amount of bacteria was increased from 0.5% to 2.5%, the sewage purification efficacy of the wetland system was significantly improved. When the amount of bacteria was increased from 2.5% to 5.0%, the purification efficacy was improved; however, the improvement was not significant. When the combined bacterium dosage was 5.0% the removal of the pollutants was improved; however, the cost was too high to make it feasible. Considering the operating costs and the efficaciousness of sewage treatment, 2.5% should be chosen as the best dosage of the mixed *Psychrotrophic* bacteria for the CW.

It can be seen in figure 4 that when the HRT was 5 days, the water temperature was 6–10°C and the dosage of combined bacterial liquid was 2.5%. As the hydraulic time was increased, the removal efficiencies of COD, NH_3_−N, ${\text{NO}}_{2}^{-}-\text{N}$, ${\text{NO}}_{3}^{-}-\text{N}$, TN and TP reached 91.8%, 91.1%, 88.0%, 93.8%, 94.8% and 95.2%, respectively, and the removal rates became 1.5, 2.0, 2.1, 1.5, 2.2 and 1.3 times higher than those of the control group. Furthermore, the average concentrations of COD, NH_3_–N, TN and TP in the effluent were 39.56, 4.21, 4.36 and 0.64 mg l^−1^, which met the first grade of Chinese national pollutant discharge standards for municipal wastewater treatment plants (GB18918-2002).

Our results showed that the removal efficiency of COD and NH_3_–N in wetland sewage was 6.4% and 6.5% higher than expected when the removal rate of the single *P. flava* WD-3 was 86.3% and 83.5% [20]. Other indicators of sewage treatment filtration did not show this level of improvement. Indeed, there were no significant differences among these indicators. Furthermore, the HRT was shortened by 5 days when the mixed *Psychrotrophic* bacteria was used in the CS (5 days versus the 10 days taken when using only *P. flava* WD-3 [20]). There this bacterial application both increased the efficacy of sewage treatment and reduced the cost of sewage purification.

Compared with one single low-temperature bacteria strain, the mixed bacteria strain better purified the sewage and shortened the HRT. In comparison, Bott & Love [21] treated aquaculture sewage with the mixed bacterial culture of *Bacillus subtilis* and photosynthetic bacteria, and found that the mixed bacterial culture had a noticeable effect on flocculation and could display synergistic properties. They found that the removal efficiencies for COD, TP and NH_3_–N were 62.35%, 66.78% and 52.60% after 48 h of treatment. Furthermore, sewage effluent transparency increased to 148.48% greater than the original, and dissolved oxygen (DO) increased 240%, compared to the original. Similarly, Moussavi & Heidarizad [22] added a mixture of bacteria to SBR directionally and found that the time of domestication and aeration was shortened, and the disposal ability and the effects of sequencing batch reactor (SBR) process were significantly improved, with a COD removal efficiency of greater than 92%. The wastewater purification abilities of *B. subtilis*, *Saccharomyces*, *Lactobacillus* and mixed strains were studied by Morikawa [23], and the results showed that the mixture of flora had a synergistic effect, improving the disposal capacity of the pollutants, with NH_3_–N and removal efficiencies reaching 93.2% and 97.8%, respectively. Therefore, a significant amount of prior research has shown that there are mutually beneficial relationships among the strains, and that mixing bacterial strains can have a synergistic effect in terms of contaminant removal efficacy. Moreover, these studies also indicated that there is potential to use these mixed bacterial strains to dispose of low-temperature sewage.

### 3.4. Wastewater treatment dynamics equation

The study of kinetics could optimize the technology and the methods associated with the biochemical disposing process. Indeed, the patterns of the microbial degradation could be predicted by establishing kinetic degradation models and simulating degradation with modern technology. The models used in the construction of the CWs were typically first-order kinetic models. This basic design equation (first-order kinetic model) has been widely applied in Australia, Europe and the USA for the prediction of bacterial treatment results. In spite of certain limitations, first-order kinetic models are still regarded as the most suitable for describing the process of degradation for pollutants in CWs. This is because calculation parameters can be worked out easily and the calculation process is simple. Jou *et al*. investigated the feasibility of using a CW to restore a heavily polluted creek, and estimated the reductions in biochemical oxygen demand (BOD) and nitrogenous biochemical oxygen demand (NBOD) using the first-order kinetic model in a laboratory wetland system, which provided some basis for using this model when designing the constructed wetlands [24].

As for the first-order kinetic equation applied in CWs, the main consideration was the relationship between the disposal load and the processing efficiency. The derivation of the model used here was based on the degradation of the matrix following the first-order reaction kinetics. The first-order kinetic model for the degradation of contaminants in CW was [25,26] as follows:

3.1 $$C_{0}=C_{e}\text{ exp}(-k_{v}\cdot t)$$

3.2 $$k_{v}=\frac{-1}{t}\text{ ln }\left(\frac{C_{0}}{C_{e}}\right).$$

In formulae (3.1) and (3.2): *k_v_*—contaminant volume removal rate constant, d^−1^; *C_e_*—influent concentration, mg · l^−1^; *C*_0_—effluent concentration, mg · l^−1^; *t*—hydraulic retention time, d.

**Figure 5.**
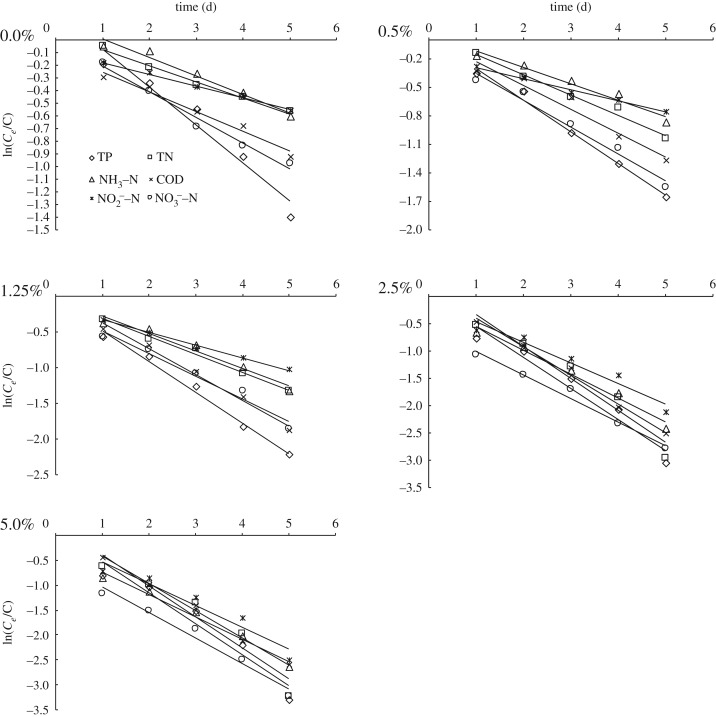
Change of pollutants with time with different dosage.

Based on the kinetic models listed above, the concentration of the pollutants in the effluent flow was denoted *C_e_* (hydraulic retention time, HRT ≤ 5 days) and the concentration of the pollutants in the influent flow was denoted *C*_0_ when the dosage differed. ln *C_e_*/*C*_0_ was taken as the ordinate and *t*(time) as the abscissa. The time curves of COD, NH_3_–N, ${\text{NO}}_{2}^{-}-\text{N}$, ${\text{NO}}_{3}^{-}-\text{N}$, TN and TP are shown in figure 5. The first-order kinetic model and *R* value of each contaminant are shown in table 3. The measurement results of COD, NH_3_–N, ${\text{NO}}_{2}^{-}-\text{N}$, ${\text{NO}}_{3}^{-}-\text{N}$, TN and TP were substituted into equation (3.2) at HRT of 5 days, and *k_v_* was calculated as shown in table 4.

**Table 3.** First-order kinetics model and the *R* value of pollutants removal in the CW.

pollution parameter	dosage (%)	First-order kinetics model	*R*^2^ value	pollution parameter	dosage (%)	first-order kinetics model	*R*^2^ value
COD	0.0	*Y* = −0.1662 *X* − 0.0965	0.9692	NH_3_–N	0.0	*Y* = −0.1439 *X* + 0.1523	0.9689
	0.50	*Y* = −0.2481 *X* + 0.0216	0.9501		0.50	*Y* = −0.1716 *X* + 0.0579	0.9613
	1.25	*Y* = −0.3561 *X* + 0.0204	0.9874		1.25	*Y* = −0.2442 *X* − 0.0290	0.9560
	2.50	*Y* = −0.5205 *X* + 0.1297	0.9895		2.50	*Y* = −0.4361 *X* − 0.1113	0.9746
	5.00	*Y* = −0.5450 *X* + 0.1331	0.9952		5.00	*Y* = −0.4449 *X* − 0.2924	0.9800
NO_3_^−^–N	0.0	*Y* = −0.2022 *X* − 0.0059	0.9809	NO_2_^−^–N	0.0	*Y* = −0.0918 *X *− 0.0829	0.9949
	0.50	*Y* = −0.2836 *X* − 0.0619	0.9730		0.50	*Y *= −0.1178 *X* − 0.1634	0.9894
	1.25	*Y* = −0.3155 *X* − 0.1673	0.9650		1.25	*Y* = −0.1760 *X* − 0.1539	0.9891
	2.50	*Y* = −0.4291 *X* − 0.5734	0.9813		2.50	*Y* = −0.3734 *X* − 0.0896	0.9469
	5.00	*Y* = −0.5085 *X* − 0.5269	0.9699		5.00	Y = −0.4353 X − 0.0822	0.9250
TN	0.0	*Y* = −0.1259 *X* + 0.0517	0.9868	TP	0.0	Y = −0.3009 *X* + 0.2262	0.9494
	0.50	*Y* = −0.2109 *X* + 0.0552	0.9823		0.50	*Y* = −0.3331 *X* + 0.0370	0.9895
	1.25	*Y* = −0.2496 *X* − 0.0630	0.9826		1.25	*Y* = −0.4280 *X* − 0.0559	0.9865
	2.50	*Y* = −0.5867 *X* + 0.2602	0.9387		2.50	*Y* = −0.5638 *X* + 0.0163	0.9490
	5.00	*Y* = −0.6186 *X* + 0.2274	0.9186		5.00	*Y* = −0.6153 *X* + 0.0806	0.9325

**Table 4.** The *k_v_* in the pseudo-first order reaction of pollutants removal in the CW.

	dosage(%)				
pollution parameter	0.00	0.50	1.25	2.50	5.00
COD	−0.1843	−0.2521	−0.3732	−0.4991	−0.5190
NH_3_–N	−0.1193	−0.1734	−0.2646	−0.4413	−0.6977
NO_2_^−^–N	−0.0692	−0.1318	−0.1802	−0.2764	−0.4979
NO_3_^−^–N	−0.1630	−0.3106	−0.3712	−0.5174	−0.3975
TN	−0.0891	−0.2065	−0.2661	−0.2848	−0.3054
TP	−0.0950	−0.3289	−0.3386	−0.3571	−0.5912

The volume degradation rate *k_v_* represents the treatment efficiency of the contaminants. Mixed *Psychrotrophic* bacterial flora had a noticeably high disposal rate for pollutants at a HRT of 5 days. Furthermore, the degradation rates of COD, NH_3_–N, ${\text{NO}}_{2}^{-}-\text{N}$, ${\text{NO}}_{3}^{-}-\text{N}$, TN and TP significantly increased as the dosage of bacteria was increased. The correlation coefficients (*R*) of each *k_v_* reached 0.9816, 0.9597, 0.9473, 0.9934, 0.9267 and 0.9183. Through the analysis of treatment efficiency for the pollutants in the CW at different dosages, it is evident that the removal kinetics of contaminants in the CW was in accordance with the first-order kinetics model.

## 4. Conclusion

(1) The nine bacterial strains, isolated from artificial wetland sediments, were cold-resistant bacteria. Following screening, a combination of the best strains (1, 4 and 5) had the highest removal efficiency for COD, NH_3_–N and TP in wastewater. Removal rates for this mixed *Psychrotrophic* bacteria strain were 86.83%, 65.95% and 56.08%, respectively. The strains 1, 4 and 5 were preliminarily identified as cold *Bacillus*, sphingolipids of *Bacillus* and *Pseudomonas*, and were named *Psychrobacter* TM-1, *Sphingobacterium* TM-2 and *Pseudomonas* TM-3.
(2) The effectiveness of the IVCW system with the mixed *Psychrotrophic* bacteria strain in winter conditions was demonstrated through the increased removal of COD, NH_3_–N, ${\text{NO}}_{2}^{-}-\text{N}$, ${\text{NO}}_{3}^{-}-\text{N}$, TN and TP. At high concentrations of organic substrate, the dosage of the mixed *Psychrotrophic* bacteria strain and the removal rate of the contaminants were positively correlated and followed a first-order rate equation. We also evaluated the effects of different bacterial dosages and determined that the optimal dosage of this mixed *Psychrotrophic* bacteria strain was 2.5% for these CW systems when considering biological effect, time, cost and resource consumption.

## Data accessibility

We include all the experimental data in the manuscript, and there is no electronic supplementary material.
